# Machine Learning-Based Sex Estimation from Computed Tomography-Derived Pelvic Morphometric Measurements in a Contemporary Turkish Population

**DOI:** 10.3390/diagnostics16182893

**Published:** 2026-09-08

**Authors:** Gokce Karaman, Ali Er, Mustafa Bozdag, Manon Hache, Siam Knecht, Sophie Colomb, Laurent Martrille, Pascal Adalian

**Affiliations:** 1 Faculty of Medicine, Department of Forensic Medicine, University of Dokuz Eylul, 35100 Izmir, Türkiye; gokce.karaman@deu.edu.tr; 2 Tepecik Training and Research Hospital, University of Health Sciences, 35180 Izmir, Türkiye; 3 Department of Forensic Medicine, CHU Dijon-Bourgogne, 21000 Dijon, France; 4 CNRS, EFS, ADES, Aix Marseille University, 13344 Marseille, France; 5 L’Équipe de Droit Pénal et de Sciences Forensiques de Montpellier (EDPFM), University of Montpellier, 34000 Montpellier, France; 6 Department of Legal Medicine, CHU Montpellier, University of Montpellier, 34000 Montpellier, France

**Keywords:** forensic anthropology, sex estimation, pelvis, computed tomography, machine learning, Turkish population

## Abstract

**Background/Objectives:** Sex estimation is a fundamental component of the forensic biological profile, and the pelvis is widely regarded as the most sexually dimorphic skeletal element. This study aimed to quantify pelvic sexual dimorphism in a contemporary Turkish sample using computed tomography (CT)-derived morphometric measurements, and to evaluate the performance of several machine learning (ML) algorithms for sex classification. **Methods:** Fourteen pelvic measurements were obtained from CT reconstructions of 201 individuals (101 males, 100 females) by a single observer; a stratified subset of 30 cases (15 males, 15 females) was remeasured by the same observer and by a second observer to assess intra- and inter-observer reliability. Four supervised ML classifiers—logistic regression, linear discriminant analysis (LDA), random forest, and support vector machine (SVM)—were trained and evaluated using stratified 10-fold cross-validation and an independent 30% hold-out test set. **Results:** Thirteen of the fourteen measurements differed significantly between sexes (*p* < 0.05). Intra-observer reliability was excellent for all 14 measurements (ICC = 0.943–0.998), and inter-observer reliability was good to excellent (ICC = 0.834–0.996), with the lowest value observed for pubic length. Cross-validated classification accuracy ranged from 98.0% (random forest) to 99.0% (logistic regression, LDA, and SVM), with corresponding cross-validated AUC values of 0.991–0.994, with logistic regression, LDA, and SVM each achieving 100% accuracy (AUC = 1.000) on the independent hold-out set (random forest: 96.7%, AUC = 0.999). Acetabular width was identified as the single most informative predictor (univariate area under the curve = 0.972), followed by acetabular height, the subpubic angle, ischial length, the transverse pelvic outlet, and the angle of the greater sciatic notch. **Conclusions:** These findings demonstrate that CT-derived pelvic morphometrics, combined with machine learning classification, provide a highly accurate and reproducible method for sex estimation in a Turkish forensic context, and support the development of population-specific standards for forensic anthropological casework, pending independent external validation; the classification and effect-size estimates reported here were, however, stable under repeated cross-validation and hyperparameter optimisation.

## 1. Introduction

Sex estimation constitutes one of the principal components of the biological profile compiled by forensic anthropologists, alongside age, stature and population affinity assessment [1]. Because subsequent estimations of age and stature are frequently derived from sex-specific standards, an erroneous sex assignment can propagate error through later stages of the identification process [1].

Among the skeletal elements available for this purpose, the os coxae is widely regarded as the most sexually dimorphic, its morphology having been shaped by the evolutionary tension between bipedal locomotion and parturition through the maternal birth canal [2]. Traditional morphoscopic methods—visual assessment of traits such as the pubic bone or the greater sciatic notch—can be rapid and accurate in experienced hands, but they remain inherently subjective, with classification error rising when the observer lacks extensive training [3,4]. Morphometric approaches, which apply statistical classification to linear and angular measurements, are comparatively less dependent on observer experience and provide a transparent, quantifiable estimate of classification accuracy suited to evidentiary requirements [5].

The integration of multidetector computed tomography (MDCT) into forensic and clinical practice has provided a further methodological advantage, allowing three-dimensional bone-rendered reconstructions to be acquired without maceration and enabling the assembly of large, contemporary reference samples [1,5], with CT-derived pelvic measurements shown to correspond closely to those obtained from direct measurement of skeletal remains [6]. Studies employing this approach have repeatedly identified the subpubic angle and the transverse pelvic diameters as among the most dimorphic single measurements, with the wide subpubic angle in females attributed in part to lateral growth of the ischia associated with obstetric adaptation [2,7]; multivariate combinations of pelvic measurements have achieved cross-validated classification accuracies approaching, or reaching, 100% [1,5].

It is nevertheless established that the magnitude and relative ranking of dimorphic pelvic traits differ across populations, reflecting the combined influence of genetic background, environment and secular change on skeletal form [7,8]. Direct comparative studies—for example between contemporary Japanese and Western Australian, or Japanese and Indonesian, samples—have documented measurable differences in absolute pelvic dimensions even where the overall pattern of sexual dimorphism is broadly conserved [9], and the application of non-population-specific standards has been shown to reduce classification accuracy [8]. These findings underscore the need for population-specific pelvic standards, an area in which data specific to the Turkish population have only recently begun to emerge. Çöllü and Bakırcı [10] recently validated the DSP2 (Diagnose Sexuelle Probabiliste V2) software using ten os coxae measurements in a Turkish sample, and Meyvaci et al. [11] applied machine learning to the subpubic angle and other palpable pelvic parameters in a separate Turkish population; both confirm strong pelvic sexual dimorphism in Turkish individuals, but neither combined, within a single study, a larger CT-derived measurement panel, a systematic comparison of multiple machine learning architectures under identical cross-validation conditions, formal intra- and inter-observer reliability assessment, and clinically usable univariate sectioning points—the combination the present study provides (compared in detail in Section 4.2).

In parallel, forensic anthropology has increasingly incorporated machine learning (ML) as a complement to classical discriminant function and logistic regression analysis. Because most ML algorithms are non-parametric, they are not constrained by the assumptions of normality and linearity underlying traditional multivariate statistics [12]. Recent studies applying random forest, support vector machine and stacked ensemble classifiers to CT-derived pelvic measurements have reported classification accuracies meeting or exceeding those obtained with conventional discriminant functions, in some cases surpassing 94–96% [9,13]. Model-agnostic interpretability tools such as SHapley Additive exPlanations (SHAP) have further been used to quantify the relative contribution of individual measurements, addressing the “black-box” criticism often raised against complex ML classifiers [9,13,14].

The present study therefore aimed (i) to quantify pelvic sexual dimorphism in a contemporary Turkish sample using CT-derived morphometric measurements, (ii) to compare the classification performance of several machine learning algorithms (logistic regression, linear discriminant analysis, random forest, and support vector machine), and (iii) to identify the pelvic parameters contributing most substantially to correct classification, in order to establish quantitatively robust, population-specific standards for Turkish forensic casework.

## 2. Materials and Methods

### 2.1. Sample Description

The present study was conducted at the Tepecik Training and Research Hospital. All medical records and CT images of patients admitted to the different clinics of the hospital between June 2014 and July 2016 were retrospectively evaluated. Cases with fracture, prior surgery, or a congenital or acquired anomaly of the pelvis were excluded from the study (*n* = 33). The final sample consisted of 201 pelvic CT examinations (101 males, 100 females) from individuals residing in İzmir, located in the western part of Turkey. Demographic information for the sample is presented in Table 1 and Table 2. The study protocol was approved by the Tepecik Training and Research Hospital Ethics Board (approval no. 2016/32/11).

### 2.2. Data Acquisition

All examinations were performed using a 64-slice CT scanner (Siemens Medical Solutions, Erlangen, Germany). A routine multidetector row computed tomography (MDCT) protocol was followed, with scanning parameters of 80 kV, 115 mAs, 1 mm slice thickness, and a 512 × 512 matrix.

**Figure 1:**
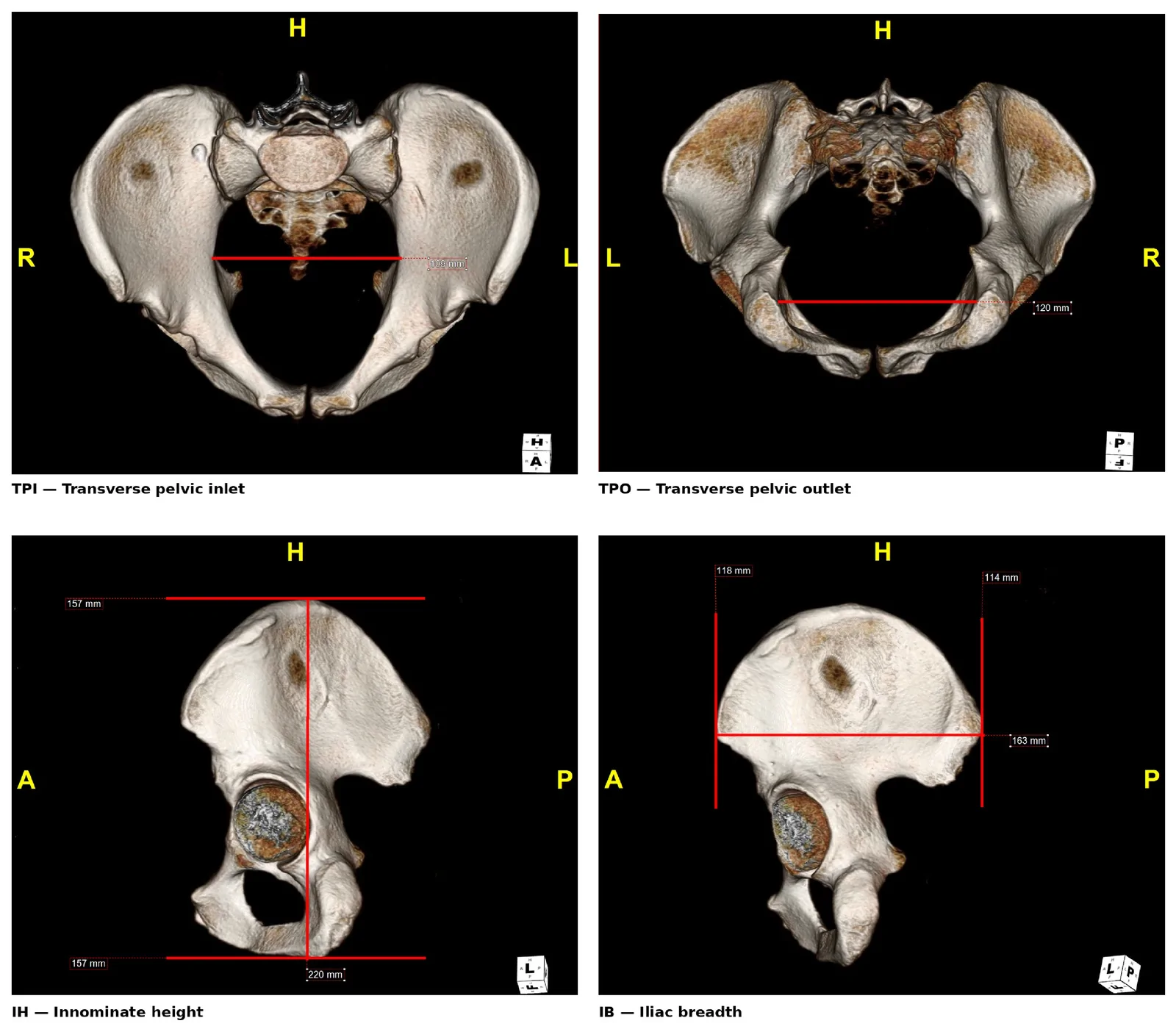
Three-dimensional CT reconstructions illustrating pelvic measurements.

**Figure 2:**
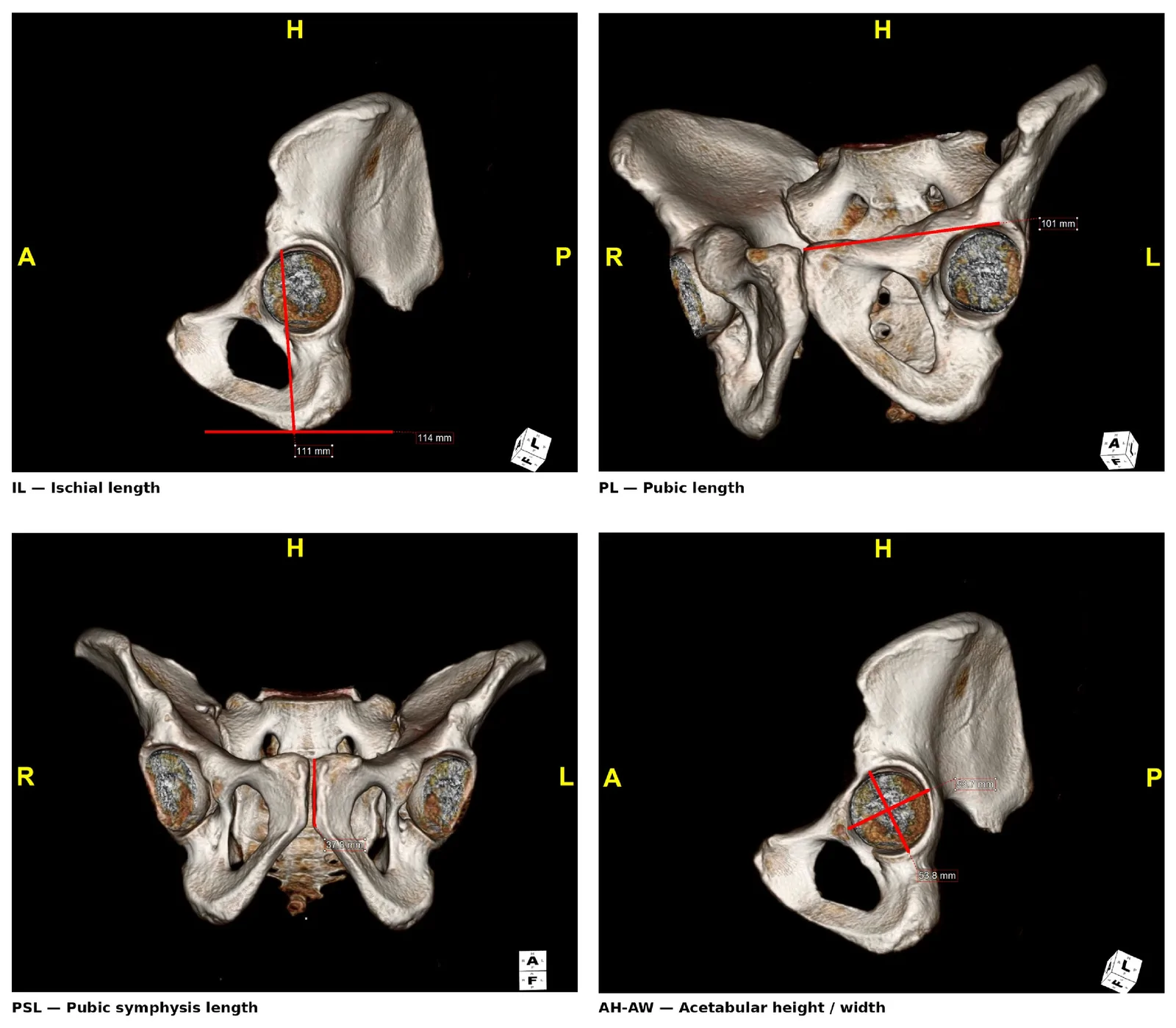
Three-dimensional CT reconstructions illustrating pelvic measurements.

**Figure 3:**
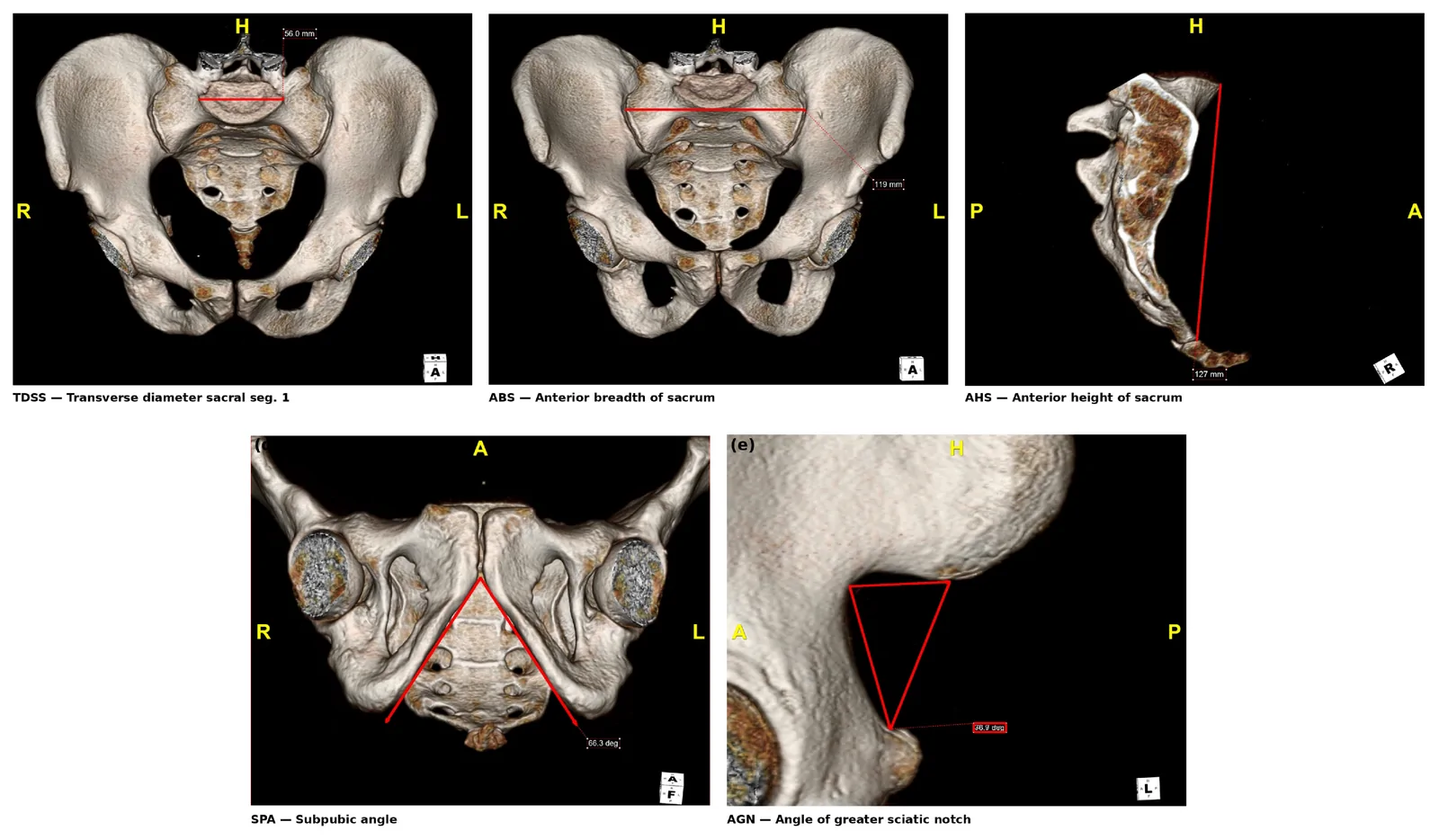
Three-dimensional CT reconstructions illustrating pelvic measurements.

In preparation for the study readings, all multidetector CT data were transferred from the archive to a workstation (Aquarius Workstation; TeraRecon, San Mateo, CA, USA) via internal network connections, providing three-dimensional post-processing options, multiplanar image reformatting (MPR), and maximum intensity projection. CT data were used to generate three-dimensional bone-rendered reconstructions of the pelvis, from which 14 morphometric measurements were obtained for each case (Figure 1, Figure 2 and Figure 3). Measurement definitions, adapted from the pelvic morphometric protocol described by Franklin et al. [1], are presented in Table 3.

### 2.3. Measurement Definitions

The 14 pelvic measurements analysed in this study—transverse pelvic inlet (TPI), transverse pelvic outlet (TPO), innominate height (IH), iliac breadth (IB), ischial length (IL), pubic length (PL), pubic symphysis length (PSL), acetabular height (AH), acetabular width (AW), transverse diameter of sacral segment 1 (TDSS), anterior breadth of the sacrum (ABS), anterior height of sacrum (AHS), subpubic angle (SPA), and angle of the greater sciatic notch (AGN)—were defined following the landmark-based protocol of Franklin et al. [1], who in turn adapted several landmark definitions from Decker et al. [15] and Buikstra and Ubelaker [16]. Full definitions and corresponding landmarks are summarised in Table 3, and representative CT reconstructions illustrating each measurement are presented in Figure 1, Figure 2 and Figure 3.

### 2.4. Measurement Reliability

All 201 cases were measured once by a single observer (R1), with 20 years of professional experience in forensic and musculoskeletal radiology, blinded to the sex and identity of each case at the time of measurement. A stratified random subset of 30 cases (15 males, 15 females) was subsequently remeasured twice: once by R1, to assess intra-observer agreement, and once by a second observer (R2, 15 years of professional experience), blinded to R1’s measurements, to assess inter-observer agreement. Measurement reliability was evaluated separately for the intra-observer and inter-observer comparisons using the intraclass correlation coefficient (ICC), calculated under a two-way mixed-effects, consistency, single-measures model (ICC[C,1]).

### 2.5. Statistical and Machine Learning Analysis

Descriptive statistics (mean, standard deviation, median, minimum, maximum, and interquartile range) were calculated for each of the 14 pelvic measurements, stratified by sex. Normality of distribution was assessed using the Shapiro–Wilk test [17]. Where both sexes met the assumption of normality, group means were compared using the independent-samples *t*-test; where this assumption was violated, the non-parametric Mann–Whitney U test was applied instead. A *p*-value < 0.05 was considered statistically significant throughout. Standardised effect sizes were additionally calculated for each measurement: Hedges’ g—the bias-corrected form of Cohen’s d, based on the pooled-variance mean difference—for measurements in which both sexes met the normality assumption, and the rank-biserial correlation derived from the Mann–Whitney U statistic otherwise; both were signed so that positive values indicate larger values in males. Multicollinearity among the 14 measurements was assessed using the variance inflation factor (VIF), obtained by regressing each measurement on the remaining 13 (ordinary least squares); a VIF above 5 was considered to indicate moderate collinearity and above 10 severe collinearity (Table 4). Because pelvic dimensions may also vary with age independently of sex, the association between age and each of the 14 measurements was evaluated using Pearson or Spearman correlation (according to normality), calculated across the pooled sample and separately within each sex; the sensitivity of the classification models to age was further assessed by repeating the 10-fold cross-validation with age included as an additional candidate predictor (Association with Age Section).

Measurement reliability was evaluated using the ICC[C,1] for each of the 14 variables, separately for the intra-observer and inter-observer comparisons within the reliability subset (n = 30). ICC values were interpreted according to the classification proposed by Koo and Li [18], whereby values below 0.50 indicate poor reliability, 0.50–0.75 moderate, 0.75–0.90 good, and above 0.90 excellent reliability.

For the machine learning analysis, all 14 pelvic measurements were entered as candidate predictor variables, with sex (male/female) as the binary outcome. Prior to model training, all variables were standardised (Z-score normalisation) to a mean of zero and unit variance. Standardisation was implemented as the first step of a scikit-learn Pipeline together with each classifier, so that in both the 10-fold cross-validation and the 70/30 hold-out analysis the mean and standard deviation used for scaling were estimated exclusively from the training portion of each fold or split and then applied, unchanged, to transform the corresponding validation or hold-out cases; at no point were validation or hold-out cases used to derive the standardisation parameters. Four supervised classification algorithms were evaluated: logistic regression, linear discriminant analysis (LDA) [19], random forest [20], and support vector machine (SVM) with a radial basis function kernel [21]. Model performance was assessed using stratified 10-fold cross-validation, with accuracy, sensitivity, specificity, positive and negative predictive values, and the area under the receiver operating characteristic curve (AUC) calculated for each classifier. To evaluate generalisability to unseen data, the dataset was additionally partitioned into a 70% training set (n = 140) and a 30% independent hold-out test set (n = 61), stratified by sex, with model performance re-evaluated on the hold-out set.

**Figure 4:**
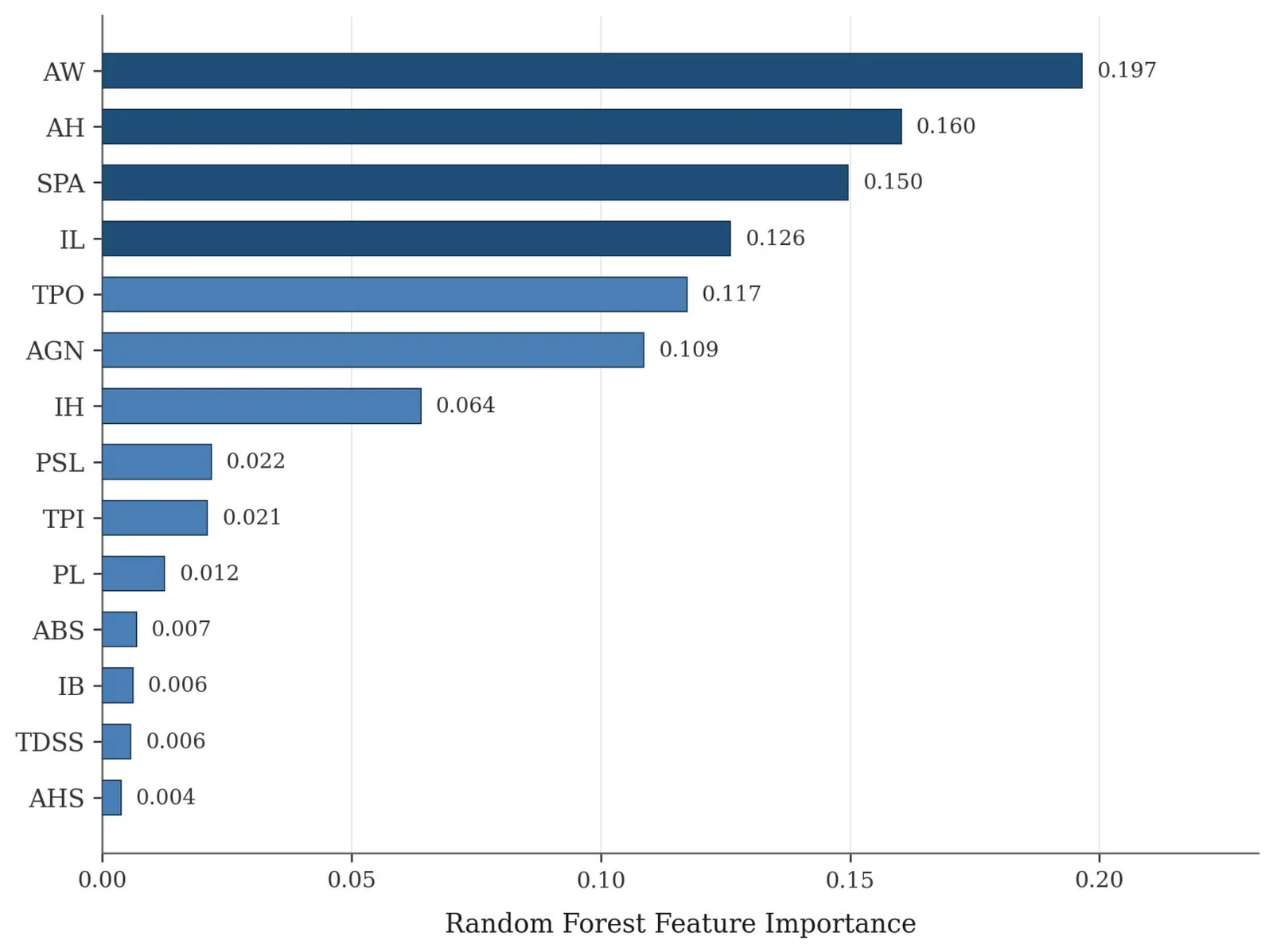
Relative contribution of the 14 pelvic measurements to sex classification (random forest feature importance).

The relative contribution of individual pelvic measurements to sex classification was assessed using random forest feature importance scores, together with standardised logistic regression coefficients and LDA discriminant function coefficients (Figure 4). For each measurement considered individually, univariate discriminative power was quantified using the AUC, and an optimal sectioning point was derived using the Youden index [22], defined as the threshold value maximising the sum of sensitivity and specificity.

Default scikit-learn hyperparameters were used for all four classifiers unless otherwise noted: logistic regression (L2 penalty, C = 1.0, lbfgs solver, max_iter = 1000); linear discriminant analysis (default singular value decomposition solver); random forest (500 trees, no maximum depth restriction, minimum samples per split = 2); and support vector machine with a radial basis function kernel (C = 1.0, gamma = “scale”). A fixed random seed (random_state = 42) was used for all stochastic components—the random forest and SVM classifiers and the stratified data splits—to ensure reproducibility; no formal hyperparameter tuning (e.g., grid search) was performed. As a supplementary sensitivity analysis, hyperparameter optimisation was performed for the random forest and support vector machine classifiers using nested cross-validation (10-fold outer loop; 3- or 5-fold inner grid search), and the stability of the primary cross-validated accuracy under repeated random partitioning was assessed using 20 independent repeats of the stratified 10-fold cross-validation, each with an independent random partition seed; these supplementary analyses (Section 3.4.4 and Section 3.4.5) are reported alongside, and do not replace, the default-hyperparameter, single-partition results that form the primary analysis throughout this manuscript. All statistical analyses and machine learning procedures were performed in Python (version 3.11), using the scikit-learn library [23] for model implementation and cross-validation.

## 3. Results

### 3.1. Sample Characteristics

The final sample comprised 201 individuals (101 males, 100 females). Mean age did not differ significantly between the sexes (males, 47.1 ± 14.3 years; females, 46.6 ± 13.9 years; independent-samples *t*-test, *p* = 0.776; Table 1), indicating that the two groups were adequately matched with respect to age and that subsequent between-sex comparisons of pelvic dimensions are unlikely to be confounded by age-related skeletal change.

### 3.2. Descriptive Statistics and Sex Differences

Descriptive statistics and the results of between-sex comparisons for the 14 pelvic measurements are presented in Table 5. Thirteen of the fourteen measurements differed significantly between males and females (*p* < 0.001 in all cases except AHS, *p* = 0.005). Only the anterior breadth of the sacrum (ABS) failed to reach statistical significance (*p* = 0.559). Although ABS did not differ significantly in univariate testing, it was retained in the multivariate machine learning models to allow for potential interactive and non-linear effects with other measurements, consistent with standard practice in ML pipelines; its low feature importance score in the random forest model (Table 6) confirms its negligible independent contribution to classification.

**Figure 5:**
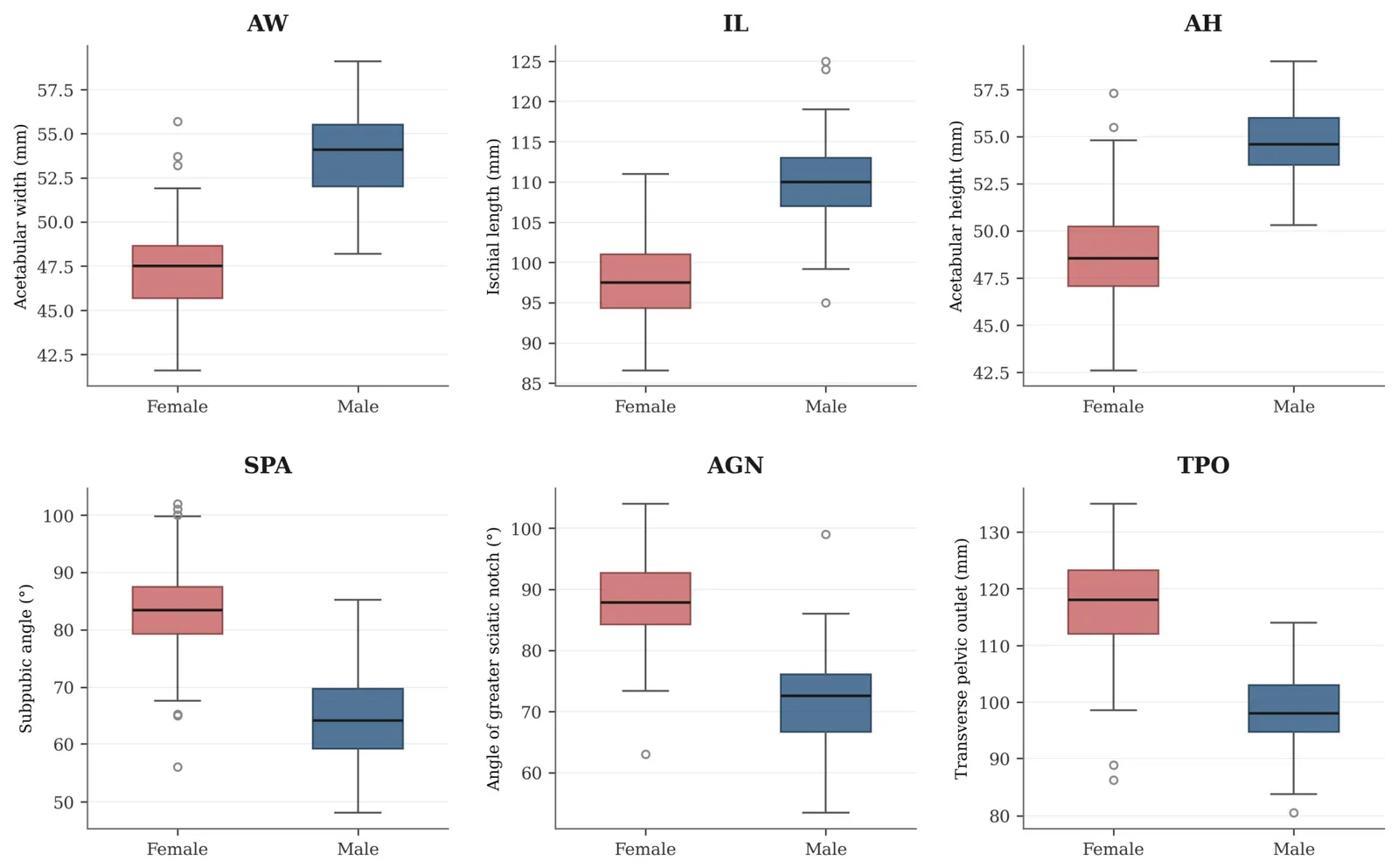
Sex differences in the six most discriminating pelvic measurements. Values are in millimetres, except SPA and AGN, which are in degrees; axis units are labelled per variable.

Consistent with the pattern described in prior CT-based pelvimetric studies [1,5], the subpubic angle (SPA) and the angle of the greater sciatic notch (AGN) were, on average, substantially wider in females (SPA: 83.4° ± 7.9° vs. 64.9° ± 7.2° in males; AGN: 87.9° ± 7.1° vs. 71.6° ± 7.3° in males), while the transverse pelvic outlet (TPO) was likewise significantly greater in females (117.2 ± 8.8 mm vs. 98.7 ± 7.5 mm in males). Conversely, measurements reflecting overall pelvic and ischial size—innominate height (IH), ischial length (IL), acetabular height (AH), and acetabular width (AW)—were consistently larger in males, in agreement with the general dimorphic pattern reported for the pelvis in other contemporary populations [1,5,9]. Sex differences for the six most discriminating measurements are illustrated in Figure 5, and for all 14 measurements in the Supplementary Figure S1.

#### Association with Age

Because pelvic morphology may vary with age independently of sex, the correlation between age and each of the 14 measurements was assessed across the pooled sample (Table 7); positive values indicate an increase with age. Five of the 14 measurements showed a statistically significant pooled correlation with age, most notably pubic symphysis length (PSL: Pearson r = 0.396, Spearman ρ = 0.391) and the transverse diameter of sacral segment 1 (TDSS: Pearson r = 0.381, Spearman ρ = 0.370), both *p* < 0.001; weaker but significant associations were also present for the transverse pelvic outlet, subpubic angle and angle of the greater sciatic notch (all |r| ≤ 0.18, *p* < 0.05). None of the six leading sex-classification predictors identified in Section 3.5 showed a pooled age correlation exceeding |r| = 0.18.

Re-running the primary 10-fold cross-validation with age added as an additional (15th) candidate predictor did not materially change classification performance for any of the four classifiers (accuracy changed by at most 0.5 percentage points, and AUC by at most 0.001; full comparison available on request), and accuracy remained stable (95.6–100%) across younger, middle and older age tertiles of the sample. Together with the age-adjusted Limitations discussion (Section 4.5), these findings indicate that age-related variation does not materially confound the present sex-classification models.

### 3.3. Measurement Reliability

Intraclass correlation coefficients (ICC[C,1]) for the intra-observer and inter-observer comparisons, calculated from a stratified 30-case reliability subset (15 males, 15 females), are presented in Table 8. Intra-observer ICC values ranged from 0.943 (pubic length, PL) to 0.998 (anterior height of sacrum, AHS), indicating excellent reliability for all 14 measurements according to the classification of Koo and Li [18]. Inter-observer ICC values ranged from 0.834 (PL; 95% CI 0.680–0.917) to 0.996 (AHS); reliability was excellent (ICC ≥ 0.90) for 13 of the 14 measurements, with pubic length showing good inter-observer reliability (ICC = 0.834), an improvement over the value obtained from the smaller, sex-imbalanced pilot subset used in an earlier stage of this study. These findings confirm that the measurement protocol was applied with a high degree of reproducibility, both by the same observer on repeated occasions and between independent observers, consistent with reliability levels reported in comparable CT-based pelvimetric studies [1,5].

### 3.4. Machine Learning Classification Performance

#### 3.4.1. 10-Fold Cross-Validation

The classification performance of the four machine learning algorithms, evaluated using stratified 10-fold cross-validation across all 201 cases, is summarised in Table 9. All four classifiers achieved high overall accuracy, ranging from 98.0% (random forest) to 99.0% (logistic regression, LDA, and SVM). The corresponding AUC values ranged from 0.991 (logistic regression) to 0.994 (random forest). Sensitivity and specificity were closely balanced across all models (98.0–99.0%), indicating that classification accuracy was not driven disproportionately by either sex.

Pairwise comparison of the classifiers’ cross-validated predictions using McNemar’s exact test revealed no statistically significant differences in classification performance between any pair of models (all *p* ≥ 0.5), confirming that the small differences in point-estimate accuracy between classifiers (e.g., 98.0% for random forest versus 99.0% for the other three models) are not statistically distinguishable given the sample size. Full pairwise results are given in Table 10.

Based on the reported classification rates and the known class distribution in the full sample (101 males, 100 females), the corresponding confusion matrices were as follows: logistic regression, LDA, and SVM each correctly classified 100 of 101 males and 99 of 100 females (1 false negative, 1 false positive); random forest correctly classified 99 of 101 males and 98 of 100 females (2 false negatives, 2 false positives). These counts clarify that the difference between 98.0% (random forest) and 99.0% (the other three classifiers) corresponds to a difference of only one additional misclassified case per sex.

#### 3.4.2. Hold-Out Validation

To evaluate model generalisability, the sample was additionally partitioned into a 70% training set (n = 140) and an independent 30% hold-out test set (n = 61). Results are presented in Table 11. Logistic regression, LDA, and SVM each achieved 100% classification accuracy (AUC = 1.000) on the independent test set, while random forest achieved 96.7% accuracy (AUC = 0.999). These results indicate that the classification models generalise well to previously unseen cases within this population sample.

To address the precision of these estimates given the modest test-set size, particularly for the three classifiers reaching 100% accuracy, sensitivity and specificity were each accompanied by Wilson 95% confidence intervals, calculated using the same method applied to accuracy. For logistic regression, LDA, and SVM: sensitivity was 100.0% [95% CI: 89.0, 100.0] (31 of 31 males correctly classified) and specificity was 100.0% [95% CI: 88.6, 100.0] (30 of 30 females correctly classified). For random forest: sensitivity was 96.8% [95% CI: 83.8, 99.4] (30 of 31 males correctly classified) and specificity was 96.7% [95% CI: 83.3, 99.4] (29 of 30 females correctly classified). These comparatively wide intervals make clear that the apparent 100% accuracy reflects the limited size of the hold-out sample and should not be interpreted as evidence of error-free classification in the broader population.

A bootstrap 95% confidence interval (2000 resamples) for hold-out AUC was likewise calculated for each classifier. For random forest, this gave AUC = 0.999 [95% CI: 0.994, 1.000]. For logistic regression, LDA, and SVM, the case-resampling bootstrap interval was degenerate at [1.000, 1.000]: because these three classifiers separated the hold-out cases perfectly (AUC = 1.000), every bootstrap resample necessarily preserves that same perfect rank-ordering, so this method cannot express the additional uncertainty that a new, unseen case might introduce. For these three classifiers, the Wilson intervals reported above for sensitivity and specificity—which treat each classified case as an independent Bernoulli trial rather than resampling from an already-separated set—provide the more informative reflection of small-sample uncertainty.

#### 3.4.3. Additional Performance Metrics

To complement accuracy, sensitivity, specificity and AUC, balanced accuracy, the F1-score and the Matthews correlation coefficient (MCC) were additionally calculated for all four classifiers under both 10-fold cross-validation and hold-out evaluation (Table 12). Because the two sexes were nearly balanced in both the full sample and the hold-out set, balanced accuracy was numerically identical to raw accuracy throughout. MCC—regarded as one of the more informative single indices for binary classification because it accounts for all four confusion-matrix cells—remained high for all classifiers (CV: 0.960–0.980; hold-out: 0.934–1.000), corroborating the accuracy- and AUC-based results reported in Section 3.4.1 and Section 3.4.2.

#### 3.4.4. Robustness to Repeated Partitioning

To assess whether the reported cross-validated accuracy depended on the particular fold partition used, the stratified 10-fold cross-validation was repeated 20 times, each with an independently generated random partition (200 fold evaluations in total per classifier), using the same default hyperparameters as the primary analysis. Mean accuracy across repeats closely matched the single-partition results reported in Table 9 for all four classifiers, with very little spread (SD ≤ 0.24 percentage points; Table 13), indicating that the reported classification performance is stable and not an artefact of a single, favourably chosen data partition.

#### 3.4.5. Hyperparameter Sensitivity Analysis

Because the primary analysis used default scikit-learn hyperparameters throughout (Section 2.5), a supplementary sensitivity analysis evaluated whether hyperparameter optimisation would materially improve performance for the two classifiers with practically tunable, non-default hyperparameters (random forest and SVM). Nested cross-validation (10-fold outer loop; grid-search inner loop) gave accuracy estimates of 98.02% (random forest) and 99.02% (SVM), and a single grid search over the full dataset identified the best-performing configurations shown in Table 14—in both cases within 0.02 percentage points of the default-hyperparameter result reported in Table 9. This indicates that the reported classification performance reflects the separability of the underlying pelvic measurements rather than favourable, arbitrarily chosen hyperparameter settings, and the default-hyperparameter results in Table 9 are retained as the primary analysis throughout this manuscript.

### 3.5. Relative Contribution of Individual Measurements

Feature importance rankings derived from the random forest model, together with standardised logistic regression coefficients and LDA discriminant function coefficients, are presented in Table 6 and illustrated in Figure 4. Acetabular width (AW) contributed most substantially to model output (random forest importance = 0.197), followed by acetabular height (AH; 0.160), subpubic angle (SPA; 0.150), ischial length (IL; 0.126), transverse pelvic outlet (TPO; 0.117), and angle of the greater sciatic notch (AGN; 0.109). Acetabular width (AW) ranked among the leading predictors across all three modelling approaches (random forest rank 1 of 14; logistic regression rank 4 of 14; LDA rank 5 of 14), indicating a comparatively robust contribution to sex classification regardless of model type. Acetabular height (AH), by contrast, was a leading predictor specifically in the random forest model (rank 2 of 14), but ranked more modestly by absolute coefficient in the logistic regression (rank 8 of 14) and LDA (rank 7 of 14) models; predictor rankings for this measurement are therefore substantially model-dependent rather than uniformly reproducible across modelling approaches. Agreement was not uniform across the full ranking, however: innominate height (IH) contributed comparatively little to the random forest model (importance = 0.064; rank 7 of 14) yet had the single largest standardised coefficient in both the logistic regression and LDA models (rank 1 of 14 in each), while the subpubic angle (SPA)—the third-ranked predictor by random forest importance—had one of the smallest LDA coefficients (rank 13 of 14). These discrepancies most likely reflect differing sensitivity to correlated and non-additive predictor relationships between the linear (logistic regression, LDA) and ensemble-based (random forest) methods, consistent with the moderate multicollinearity noted among several acetabular and pelvic-outlet measurements (Section 4.5).

**Figure 6:**
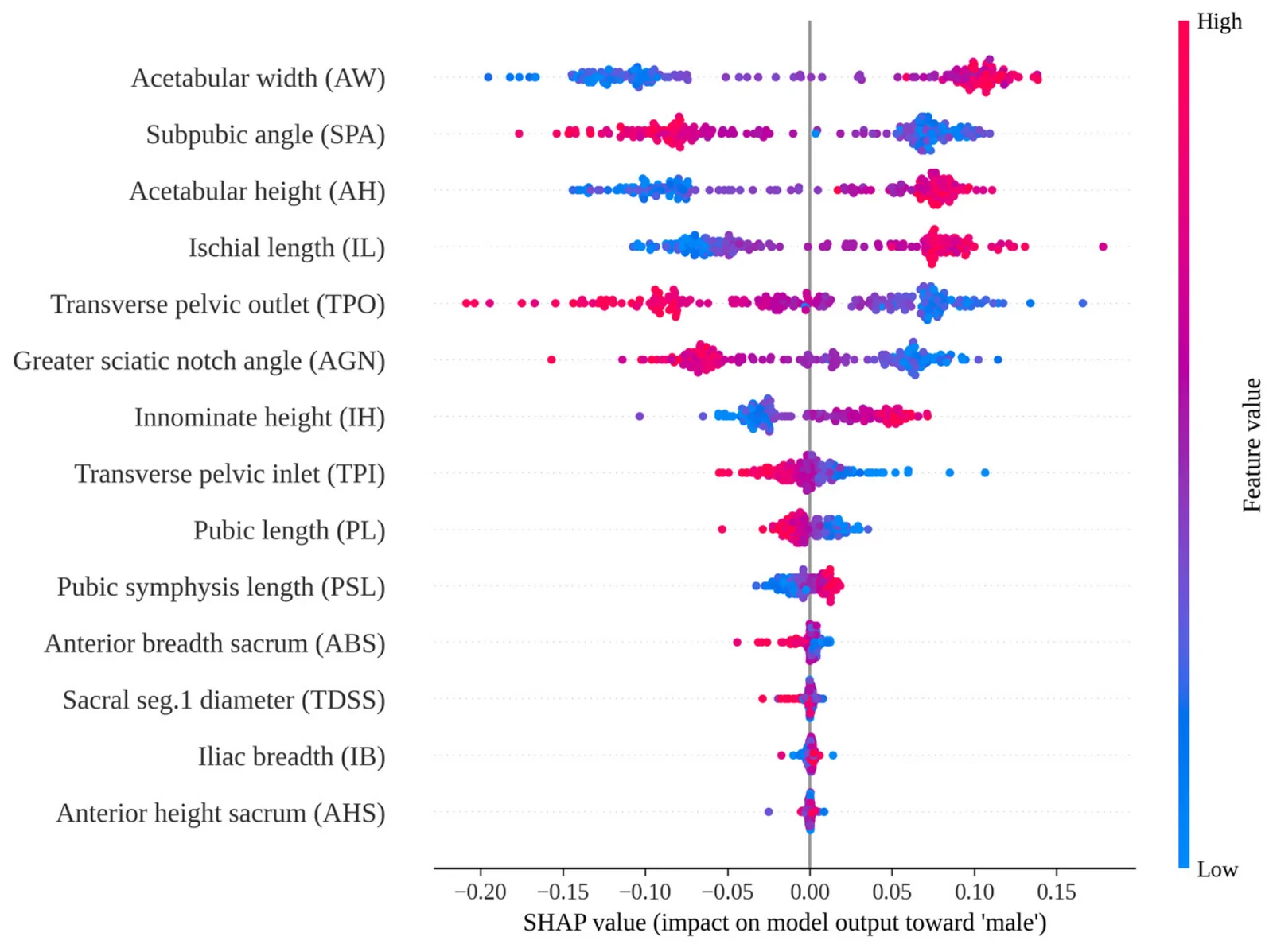
SHAP summary plot for the random forest classifier. Each point is one individual; position along the *x*-axis shows the SHAP value (impact on the model output toward classification as male) for that measurement in that individual, and colour shows the individual’s raw value for the measurement (red = higher, blue = lower).

As a complementary, model-agnostic interpretability check, SHapley Additive exPlanations (SHAP) values were computed for the random forest classifier (TreeExplainer; same hyperparameters and full N  =  201 sample as Figure 4 and Table 6). The resulting summary plot (Figure 6) confirms acetabular width as the leading contributor to classification toward the male class, with larger values of acetabular width, height, ischial length and the transverse pelvic outlet, and smaller values of the subpubic angle and the angle of the greater sciatic notch, pushing predictions toward male—directionally consistent with the between-sex differences reported in Table 5—and reproduces the same broad ranking as the random forest importance scores in Table 6, with minor reordering among the closely clustered second-tier predictors (acetabular height and the subpubic angle) that reflects ordinary numerical sensitivity rather than a substantive discrepancy.

### 3.6. Univariate Discrimination and Sectioning Points

The univariate discriminative capacity of each pelvic measurement, together with the corresponding Youden-index sectioning point, is presented in Table 15. Acetabular width (AW) demonstrated the highest single-variable discriminative power (AUC = 0.972), correctly classifying 92.5% of individuals at a sectioning point of 50.9 mm, above which classification favoured the male sex. This was followed by ischial length (IL; AUC = 0.961, sectioning point 104.0 mm), acetabular height (AH; AUC = 0.958, sectioning point 51.2 mm), subpubic angle (SPA; AUC = 0.955, sectioning point 74.9°), and angle of the greater sciatic notch (AGN; AUC = 0.950, sectioning point 80.8°). The lowest univariate discriminative power was observed for anterior breadth of the sacrum (ABS; AUC = 0.517), consistent with its non-significant between-sex difference in the descriptive analysis (Section 3.2).

To assess whether these sectioning points were overly optimistic when estimated and evaluated on the same sample, we performed a bootstrap validation (1000 resamples) in which the Youden-optimal cutpoint for each variable was re-derived on each resample and then evaluated on the corresponding out-of-bag cases. The resulting “optimism”—the difference between the apparent, same-sample accuracy and the out-of-bag accuracy—was modest for all 14 variables, ranging from approximately 1 to 3 percentage points (e.g., 1.6 points for acetabular width, the strongest univariate predictor), indicating that the reported sectioning-point accuracies are not substantially inflated by same-sample estimation. Full per-variable results are given in Table 16.

## 4. Discussion

The present study demonstrates that the pelvis of a contemporary Turkish sample exhibits pronounced sexual dimorphism, with 13 of the 14 morphometric parameters examined differing significantly between males and females, and that this dimorphism can be exploited by supervised machine learning classifiers to estimate sex with near-perfect accuracy. These findings are consistent with, and in several respects exceed, those previously reported for other contemporary populations examined using comparable CT-based pelvimetric protocols [1,5,9].

### 4.1. Pattern and Magnitude of Pelvic Dimorphism in the Turkish Sample

The pattern of dimorphism observed here—wider subpubic angle (SPA), wider angle of the greater sciatic notch (AGN), and a broader transverse pelvic outlet (TPO) in females, contrasted with generally larger acetabular and ischial dimensions in males—mirrors the pattern reported in both a contemporary Western Australian sample [1] and a contemporary Japanese sample [5], and has consistently been interpreted as a functional consequence of the competing selective pressures of bipedal locomotion and parturition [2,7]. However, the absolute magnitude of specific measurements varied considerably between these populations. The mean subpubic angle for Turkish females in the present sample (83.4° ± 7.9°) was distinctly narrower than that reported for Japanese females (112.7° ± 10.1°) [5] and for Western Australian females (89.4° ± 7.3°) [1], and also somewhat narrower than values reported for South African white and American white female samples (93.9° and 88.4°, respectively) [24,25], a pattern of pronounced subpubic angle dimorphism also documented in Greek and South African black samples [26,27]. Turkish male SPA values (64.9° ± 7.2°) were, by contrast, closer to those reported for American white males (63.7°) than to Western Australian (69.0°) or Japanese (74.8°) males [1,5,24]. A broadly comparable pattern was observed for the angle of the greater sciatic notch, where Turkish female values (87.9° ± 7.1°) exceeded those reported for both the Western Australian (84.0°) and Japanese (82.5°) samples [1,5]. These discrepancies reinforce the well-established observation that, although the overall pattern of pelvic sexual dimorphism appears broadly conserved across human populations, the absolute expression of individual dimorphic traits is population-specific [8,9], and confirm that formulae or sectioning points derived from non-Turkish samples would be unlikely to transfer accurately to a Turkish forensic context.

A further population-specific observation concerns the single measurement that failed to reach statistical significance between sexes. In the present sample, this was the anterior breadth of the sacrum (ABS, *p* = 0.559). This departs from both the Western Australian sample, in which pubic length was the principal non-dimorphic variable [1], and the Japanese sample, in which the anterior upper spinal breadth of the pelvis (a measurement not included in the present protocol) showed no significant sex difference [5]. That a different single measurement loses discriminatory value in each population that has been examined lends further support to the view that the sacrum, in particular, displays comparatively unstable and population-dependent sexual dimorphism, a conclusion also reached in CT-based analyses of the isolated sacrum, where classification accuracy has consistently remained below 70–80% regardless of population [1,28].

### 4.2. Comparative Classification Performance

The classification accuracy achieved through the machine learning models in the present study—98.0–99.0% under 10-fold cross-validation and up to 100% on an independent hold-out test set—is at the upper end of, and in several instances exceeds, accuracy rates reported using both classical discriminant function analysis and more recent machine learning approaches applied to the pelvis. Classical stepwise discriminant function analysis of the complete pelvis has previously achieved 98.1% accuracy in a Japanese sample [5] and 100% accuracy using a ten-variable stepwise function in a Western Australian sample [1], while a single-variable model based on the subpubic angle alone achieved 93.2–98.1% accuracy in these respective populations [1,5]. In the present study, no single variable matched this level of accuracy in isolation—acetabular width, the strongest univariate predictor, achieved an AUC of 0.972 and 92.5% classification accuracy at its optimal sectioning point—but the multivariate machine learning models incorporating all 14 measurements substantially exceeded these univariate benchmarks.

Direct comparison with other recently published machine learning applications to pelvic sex estimation is similarly favourable. In a contemporary Black South African sample, a stacking ensemble combining Extra Trees, random forest and XGBoost as base learners achieved a pelvic classification accuracy of 96.1%, itself a substantial improvement over the best individual base classifier (Extra Trees, 93.2%) and over the logistic regression and linear discriminant analysis benchmarks evaluated in that study (both 90.4%) [13]. The present study’s cross-validated accuracy of 98.0–99.0%, achieved without a stacking architecture, suggests that the Turkish pelvic sample may display a degree of sexual dimorphism at least comparable to, and possibly exceeding, that reported for the South African sample, although differences in sample size, age distribution, and the specific measurement sets used preclude a fully direct comparison. It is also notable that a closely related application of random forest and support vector machine classifiers to pelvic measurements—in that instance for the discrimination of population affinity between Japanese and Indonesian samples rather than sex—achieved broadly similar accuracy levels (94.1–96.4%) [9], suggesting that ensemble and margin-based classifiers are, more generally, well suited to the moderate-dimensionality, moderately correlated feature sets typical of pelvic osteometric data.

Two recent studies have specifically examined pelvic sex estimation in Turkish populations, providing a valuable direct point of comparison for the present findings. Çöllü and Bakırcı [10] validated the DSP2 (Diagnose Sexuelle Probabiliste V2) probabilistic software using ten morphometric measurements of the os coxae obtained from CT reconstructions in 200 Turkish individuals (100 male, 100 female), reporting a sexing rate of 98.5% and an accuracy rate of 100% at a posterior probability threshold of 0.95 when all ten variables were used. These figures are strikingly consistent with the 98.0–99.0% cross-validated accuracy obtained in the present study using an independently developed set of machine learning classifiers. Notably, nine of the ten DSP2 variables demonstrated significant sexual dimorphism in their sample, with only the spino-auricular length (SA) failing to reach significance—a pattern broadly analogous to the present study, in which all measurements except the anterior breadth of the sacrum (ABS) were significantly dimorphic, even though the two non-significant variables are anatomically distinct. In a separate Turkish sample, Meyvaci et al. [11] applied machine learning algorithms to the subpubic angle and other palpable pelvic parameters for sex estimation, further supporting the applicability of ML-based approaches to pelvic sexing in this population. Taken together with the present findings, these studies indicate that the Turkish pelvis exhibits a level of sexual dimorphism and an amenability to both probabilistic and machine learning-based classification, comparable to that reported in several other contemporary populations [1,5,9,13], and that the two methodological traditions—landmark-based probabilistic software such as DSP2 and multivariate supervised classifiers—produce convergent, mutually reinforcing evidence for this population. Relative to these two Turkish studies specifically, the present work additionally provides a larger 14-variable CT-derived measurement panel (versus the 10 DSP2 variables used by Çöllü and Bakırcı [10]), a systematic within-study comparison of four machine learning architectures evaluated under identical cross-validation and hold-out conditions, formal intra- and inter-observer reliability assessment (Section 3.3), and univariate sectioning points suited to fragmentary remains (Table 15)—together the combination of features that distinguishes the present study within the Turkish pelvic sex-estimation literature.

A noteworthy observation in the present study is the elevated relative importance of acetabular width and height as primary sex discriminators. This finding diverges from traditional morphometric frameworks, which have historically prioritised the subpubic angle and the greater sciatic notch as the most robust indicators of sexual dimorphism [1,5]. In classical discriminant function analysis (DFA), the subpubic angle has long been considered a primary landmark due to its direct evolutionary association with the obstetric requirements of the female pelvis [2].

The comparatively greater weight assigned to acetabular dimensions by the random forest model—an ensemble, non-linear classifier—relative to the linear models (logistic regression, LDA) raises the possibility that acetabular-based sex differences in this sample are not purely additive and may involve interactions with other pelvic measurements that linear models are less able to capture. RBF-SVM, although likewise non-linear, is not an ensemble method, and because no SVM-specific feature importance or interpretability analysis was performed in the present study, this observation is restricted to the random-forest results and should not be extended to SVM without dedicated interpretability analysis for that classifier. This interpretation should be treated as a hypothesis rather than a demonstrated finding: the feature importance comparison in Figure 4 shows a difference in predictor weighting between model types, but the present analyses did not include a formal test for non-linear interactions (e.g., explicit interaction terms, partial dependence analysis, or SHAP interaction values), and Figure 4 alone does not constitute direct evidence that such interactions were captured.

We propose, as a hypothesis for future investigation rather than an established mechanism, that the acetabulum’s high predictive power may relate to its central position within the pelvic girdle: its dimensions are anatomically linked to ischial and pubic morphology through developmental and mechanical relationships, and it is plausible that some of this linkage is non-additive. Testing this hypothesis directly would require dedicated interaction and interpretability analyses (e.g., SHAP interaction values, partial dependence plots) in a sample large enough to support them; the present study was not designed to, and does not, establish such interactions as a demonstrated mechanism.

### 4.3. Relative Contribution of Individual Measurements

Feature importance analysis in the present study identified acetabular width and acetabular height as the two most influential predictors, followed by the subpubic angle, ischial length, transverse pelvic outlet, and the angle of the greater sciatic notch. This finding is only partly concordant with earlier discriminant-function-based studies, which have generally identified the subpubic angle and transverse pelvic outlet as the most powerful discriminators of sex in both Western Australian and Japanese samples [1,5]. It is, however, strikingly consistent with the feature importance rankings reported in the South African machine learning study, in which acetabular diameter emerged as the single most influential SHAP-ranked predictor for pelvic sex classification, ahead of total pelvic height and the greater sciatic notch [13]. Taken together, these findings raise the possibility that acetabular dimensions carry more classification-relevant information than has traditionally been emphasised in discriminant-function-based pelvimetric literature, and that machine learning approaches—which do not presuppose a linear or additive relationship between predictors and outcome—may be more sensitive to this contribution than classical stepwise methods, which tend to preferentially select variables with the single largest F-statistic (typically the subpubic angle or transverse pelvic outlet) before smaller but complementary sources of variance are considered [1,5].

### 4.4. Measurement Reliability

The single exception was pubic length (PL), for which inter-observer reliability was comparatively lower, though still good (ICC = 0.834), while its intra-observer reliability remained excellent (ICC = 0.943); this pattern suggests that PL identification is more sensitive to inter-observer differences in landmark placement than to within-observer variability, and that particular care in standardising the PL landmark definition may be warranted for future studies using this protocol.

### 4.5. Limitations

Several limitations should be acknowledged. First, the sample was drawn from a single institution in İzmir and may not be fully representative of the broader Turkish population, which displays considerable regional heterogeneity. Second, moderate multicollinearity was present among several of the acetabular and pelvic-outlet measurements (variance inflation factors up to 7.1 for acetabular width; Table 4); although no variable exceeded the conventional threshold of 10, this collinearity should be borne in mind when interpreting the relative ranking of individual feature importance, particularly for the logistic regression and LDA coefficients. Third, although mean age did not differ significantly between the male and female groups in this sample, pelvic morphology is known to undergo continuous, sex-specific age-related remodelling throughout adulthood [29, 30]; age showed a statistically significant pooled correlation with 5 of the 14 measurements (most notably pubic symphysis length and the sacral segment-1 diameter, both r_s_ ≈ 0.37–0.39), and including age as an additional candidate predictor did not materially change cross-validated classification performance (Association with Age Section, Table 7), suggesting that age-related variation does not substantially confound the present sex-classification models. Nonetheless, the present cross-sectional design did not allow the longitudinal trajectory of age-related remodelling to be modelled explicitly. Fourth, as with all population-specific discriminant models, the classification accuracies and sectioning points reported here should not be assumed to generalise to other populations without independent validation [8,9]. Fifth, the reported hold-out accuracy, although accompanied by a 95% confidence interval to guard against overinterpretation of a single partition, was nonetheless derived from one stratified 70/30 split of the data; repeated or externally collected hold-out samples would provide a more robust estimate of generalisability. Relatedly, although the primary analyses used default classifier hyperparameters throughout, a supplementary nested-cross-validation grid search for random forest and support vector machine found no material improvement over the default configuration (Section 3.4.5, Table 14), and repeated stratified 10-fold cross-validation across 20 independent partitions confirmed that accuracy was stable to within approximately ±0.2–0.5 percentage points (Section 3.4.4, Table 13), together suggesting that the reported performance is neither an artefact of arbitrarily chosen hyperparameters nor of a single fortunate data partition. Sixth, because the machine learning models were trained on Z-score-standardised measurements, practical application by other researchers or practitioners requires either use of the same standardisation parameters reported here or re-derivation of the models’ raw, unstandardised coefficients; a user-facing calculation tool would facilitate wider forensic adoption. Seventh, formal calibration of the predicted class probabilities (e.g., calibration curves or Brier scores) was not assessed; although this does not affect the reported hard-classification accuracies, calibrated probabilities would be relevant if posterior probabilities rather than binary classifications are required in casework. Finally, and most importantly, as in comparable studies [1,5,13], the reported cross-validated and hold-out accuracies, while methodologically rigorous and stable under repeated partitioning (Section 3.4.4), were obtained entirely from a single-institution dataset in which model development and evaluation shared the same source population; true generalisability cannot be established until these models and sectioning points are evaluated in an independently collected Turkish cohort from a different institution, and the models should be regarded as provisional, population- and centre-specific reference standards pending such external validation.

### 4.6. Forensic Implications

Despite these limitations, the present findings support the forensic utility of CT-derived pelvic morphometrics for sex estimation in a Turkish context. The identification of robust single-variable sectioning points, particularly for acetabular width, ischial length and the subpubic angle, may be of particular value in cases involving fragmentary or incomplete pelvic remains, where multivariate assessment of the complete pelvis is not possible [1]. More broadly, the performance of the machine learning classifiers evaluated here adds to a growing body of evidence that non-parametric, ensemble-based and margin-based approaches can equal or exceed the performance of traditional discriminant function analysis for pelvic sex estimation [9,13], while offering a methodologically transparent and reproducible framework suited to the evidentiary standards required in medicolegal practice.

## 5. Conclusions

The present study demonstrates that the pelvis of a contemporary Turkish population exhibits marked sexual dimorphism, with 13 of 14 CT-derived morphometric parameters differing significantly between males and females, and confirms that this dimorphism can be exploited with excellent precision using supervised machine learning classification. All four algorithms evaluated—logistic regression, linear discriminant analysis, random forest, and support vector machine—achieved cross-validated classification accuracies of 98.0–99.0%, with logistic regression, linear discriminant analysis and support vector machine each reaching 100% accuracy on an independent hold-out test set. Acetabular width, acetabular height, the subpubic angle, ischial length, the transverse pelvic outlet, and the angle of the greater sciatic notch were identified as the most informative predictors, a ranking that both corroborates and, in the relative prominence it assigns to acetabular dimensions, extends findings previously reported for other populations examined using comparable methodology [1,5,13].

The excellent intra-observer (ICC = 0.943–0.998) and good-to-excellent inter-observer (ICC = 0.834–0.996) reliability achieved for the 14 measurements supports the reproducibility of the protocol, while the population-specific differences observed relative to Western Australian, Japanese, and South African samples [1,5,9,13] reinforce the principle that pelvic sex-estimation standards should not be applied outside the population in which they were developed [8,9]. The models and sectioning points presented here are intended to provide a quantitatively robust, population-specific reference for forensic anthropological casework involving Turkish skeletal remains and may be of particular value where only a single measurement or a fragmentary pelvis is available for analysis. Future work incorporating larger and geographically more diverse Turkish samples, together with independent external validation, would further strengthen the applicability of these findings to routine forensic practice.

## Figures and Tables

**Table 1:** Demographic characteristics of the study sample.

Variable	Male (n = 101)	Female (n = 100)	Test Statistic	*p*-Value
Age (years), mean ± SD	47.1 ± 14.3	46.6 ± 13.9	t = 0.285	0.776
Age (years), range (min–max)	18–77	18–86	–	–

SD, standard deviation. Age did not differ significantly between sexes (independent-samples *t*-test, *p* = 0.776).

**Table 2:** Age distribution by sex and 10-year age group (*N* = 201).

Age Group (Years)	Male (N)	Female (N)	Total (N)
18–27	11	9	20
28–37	18	20	38
38–47	17	24	41
48–57	28	24	52
58–67	19	15	34
68–77	8	7	15
78–87	0	1	1

The 78–87 age band contains a single female case.

**Table 3:** Definitions of the 14 pelvic measurements used in the present study.

Abbrev.	Measurement	Definition	Landmarks
TPI	Transverse pelvic inlet	Widest medio-lateral distance across the plane formed by the sacral promontory and the superior point of the pubic symphysis	pi–pi
TPO	Transverse pelvic outlet	Widest medio-lateral distance across the plane formed by the coccyx, ischial tuberosity, and inferior point of the pubic symphysis	po–po
IH	Innominate height	Distance from the most superior point of the iliac crest to the most inferior central point of the ischial tuberosity	sic–it
IB	Iliac breadth	Distance from the anterior superior to the posterior superior iliac spine	asic–psis
IL	Ischial length	Distance from the superior acetabular border to the most inferior central point of the ischial tuberosity	sab–it
PL	Pubic length	Distance from the superior acetabular border to the most anterosuperior point of the pubic symphysis	sab–sps
PSL	Pubic symphysis length	Distance between the most superior and most inferior points of the pubic symphysis	sps–ips
AH	Acetabular height	Distance between the superior and inferior borders of the acetabulum	sab–iab
AW	Acetabular width	Distance between the medial and lateral borders of the acetabulum	mab–lab
TDSS	Transverse diameter of sacral segment 1	Distance between the two most lateral points of the first sacral segment	sls–sls
ABS	Anterior breadth of the sacrum	Maximum transverse distance across the sacrum at the anterior projection of the auricular surface	sw–sw
AHS	Anterior height of sacrum	Distance between the sacral promontory and the sacro-coccygeal border	scp–scb
SPA	Subpubic angle	Angle formed by the inferior pubic symphysis and the ischiopubic ramus bilaterally	ipr–ips–ipr
AGN	Angle of greater sciatic notch	Angle formed by the posterior inferior iliac spine, the deepest point of the greater sciatic notch, and the ischial spine	piis–gsn–is

Measurement definitions and landmark abbreviations adapted from Franklin et al. [1], with several landmark definitions originally following Decker et al. [15] and Buikstra and Ubelaker [16].

**Table 4:** Variance inflation factors (VIF) for the 14 pelvic morphometric measurements used in the machine learning models.

Variable	Variance Inflation Factor (VIF)
AW	7.09
AH	6.29
TPO	6.04
IL	5.91
SPA	5.32
IH	4.65
TPI	3.48
AGN	2.39
ABS	2.17
IB	2.02
PSL	1.76
PL	1.59
AHS	1.34
TDSS	1.25

VIF was calculated by regressing each measurement on the remaining 13 measurements (ordinary least squares) and computing VIF = 1/(1 − R^2^). A VIF exceeding 5 indicates moderate multicollinearity; a VIF exceeding 10 is conventionally considered severe. No variable exceeded the threshold of 10.

**Table 5:** Descriptive statistics and between-sex comparison for the 14 pelvic measurements.

Variable	Male Mean ± SD	Male Median (Range)	Female Mean ± SD	Female Median (Range)	Test	*p*	Effect Size
TPI	126.04 ± 7.93	126.0 (108.0–147.0)	133.46 ± 7.22	133.5 (115.0–150.0)	*t*-test	<0.001	g = −0.97
TPO	98.67 ± 7.53	98.0 (80.5–114.0)	117.18 ± 8.85	118.0 (86.2–135.0)	MWU	<0.001	r = −0.88
IH	218.22 ± 9.73	217.0 (194.0–245.0)	199.68 ± 8.05	199.0 (182.0–218.0)	*t*-test	<0.001	g = +2.07
IB	158.94 ± 7.62	159.0 (142.0–181.0)	154.22 ± 7.15	154.0 (138.0–173.0)	*t*-test	<0.001	g = +0.64
IL	109.85 ± 5.15	110.0 (95.0–125.0)	97.53 ± 4.77	97.5 (86.6–111.0)	*t*-test	<0.001	g = +2.47
PL	97.19 ± 5.61	97.0 (85.7–110.0)	99.90 ± 4.29	100.0 (83.8–109.0)	MWU	<0.001	r = −0.31
PSL	37.33 ± 4.15	37.1 (26.4–49.1)	32.72 ± 3.43	32.6 (25.2–42.4)	*t*-test	<0.001	g = +1.20
AH	54.70 ± 2.04	54.6 (50.3–59.0)	48.79 ± 2.53	48.5 (42.6–57.3)	MWU	<0.001	r = +0.92
AW	53.86 ± 2.38	54.1 (48.2–59.1)	47.45 ± 2.29	47.5 (41.6–55.7)	MWU	<0.001	r = +0.94
TDSS	53.15 ± 4.85	53.0 (42.4–72.0)	50.64 ± 5.66	50.0 (40.0–65.0)	MWU	<0.001	r = +0.29
ABS	117.33 ± 6.87	117.0 (101.0–133.0)	117.88 ± 6.54	117.0 (104.0–137.0)	*t*-test	0.559	g = −0.08
AHS	121.41 ± 11.68	123.0 (99.1–150.0)	116.64 ± 12.32	117.0 (80.1–146.0)	*t*-test	0.005	g = +0.40
SPA	64.87 ± 7.22	64.2 (48.1–85.2)	83.38 ± 7.90	83.5 (56.0–102.0)	MWU	<0.001	r = −0.91
AGN	71.55 ± 7.25	72.6 (53.5–99.0)	87.89 ± 7.07	87.8 (63.0–104.0)	*t*-test	<0.001	g = −2.27

Values are mean ± SD and median (range), in millimetres or degrees (for SPA and AGN). MWU, Mann–Whitney U test. All measurements differed significantly between sexes (*p* < 0.05) except anterior breadth of the sacrum (ABS, *p* = 0.559). Effect size is Hedges’ g (bias-corrected Cohen’s d) for measurements meeting the normality assumption in both sexes, or the rank-biserial correlation r otherwise (Section 2.5); both are signed so that positive values indicate larger values in males. Conventional benchmarks: |g| ≈ 0.2/0.5/0.8 and |r| ≈ 0.1/0.3/0.5 correspond to small/medium/large effects.

**Table 7:** Correlation between age and each of the 14 pelvic measurements (pooled sample, N  =  201).

Variable	Pearson r	*p*	Spearman ρ	*p*
TPI	+0.108	0.128	+0.074	0.293
TPO	−0.159	0.024	−0.172	0.015 *
IH	+0.006	0.934	−0.012	0.866
IB	+0.096	0.176	+0.096	0.175
IL	+0.089	0.209	+0.084	0.234
PL	+0.028	0.694	−0.005	0.949
PSL	+0.396	<0.001	+0.391	<0.001 *
AH	+0.058	0.414	+0.070	0.321
AW	+0.068	0.336	+0.075	0.290
TDSS	+0.381	<0.001	+0.370	<0.001 *
ABS	+0.078	0.270	+0.079	0.266
AHS	−0.064	0.363	−0.074	0.300
SPA	−0.154	0.029	−0.184	0.009 *
AGN	−0.138	0.050	−0.155	0.028 *

Pearson r assumes a linear association; Spearman ρ is the rank-based alternative used throughout as the primary estimate given non-normality in several variables (Section 2.5). * *p* < 0.05 for at least one coefficient.

**Table 6:** Relative importance of the 14 pelvic measurements for sex classification.

Variable	Random Forest Importance	Logistic Regression Std. Coefficient	LDA Coefficient
AW	0.197	0.873	2.181
AH	0.160	0.468	1.728
SPA	0.150	−0.432	0.108
IL	0.126	1.051	2.147
TPO	0.117	−0.487	−3.561
AGN	0.109	−0.766	−3.738
IH	0.064	1.957	6.144
PSL	0.022	0.074	0.068
TPI	0.021	−0.653	−0.688
PL	0.013	−1.048	−2.984
ABS	0.007	−0.417	−1.289
IB	0.006	−0.297	−0.624
TDSS	0.006	−0.136	−0.986
AHS	0.004	0.269	−0.229

Ranked by random forest importance score (500 trees, n = 201). LDA, linear discriminant analysis.

**Table 8:** Intra-observer and inter-observer reliability (intraclass correlation coefficient) for the 14 pelvic measurements.

Variable	Intra ICC(C,1)	Intra 95% CI	Inter ICC(C,1)	Inter 95% CI	*p*
TPI	0.996	[0.99, 1.00]	0.990	[0.98, 1.00]	<0.001
TPO	0.996	[0.99, 1.00]	0.994	[0.99, 1.00]	<0.001
IH	0.985	[0.97, 0.99]	0.982	[0.96, 0.99]	<0.001
IB	0.977	[0.95, 0.99]	0.965	[0.93, 0.98]	<0.001
IL	0.989	[0.98, 0.99]	0.982	[0.96, 0.99]	<0.001
PL	0.943	[0.88, 0.97]	0.834	[0.68, 0.92]	<0.001
PSL	0.984	[0.97, 0.99]	0.983	[0.97, 0.99]	<0.001
AH	0.983	[0.96, 0.99]	0.985	[0.97, 0.99]	<0.001
AW	0.986	[0.97, 0.99]	0.985	[0.97, 0.99]	<0.001
TDSS	0.980	[0.96, 0.99]	0.966	[0.93, 0.98]	<0.001
ABS	0.992	[0.98, 1.00]	0.995	[0.99, 1.00]	<0.001
AHS	0.998	[1.00, 1.00]	0.996	[0.99, 1.00]	<0.001
SPA	0.995	[0.99, 1.00]	0.992	[0.98, 1.00]	<0.001
AGN	0.993	[0.98, 1.00]	0.990	[0.98, 1.00]	<0.001

ICC calculated using a two-way mixed-effects, consistency, single-measures model (ICC[C,1]) from a stratified reliability subset of 30 cases (15 males, 15 females). Intra-observer ICC compares the original measurement with a repeat measurement by the same observer; inter-observer ICC compares the original measurement with an independent measurement by a second observer. Reliability was excellent (ICC ≥ 0.90) for all measurements except inter-observer pubic length (PL; ICC = 0.834), which was good [18].

**Table 9:** Classification performance of four machine learning algorithms under stratified 10-fold cross-validation (n = 201).

Model	Accuracy	AUC	Sensitivity (Male)	Specificity (Female)	PPV	NPV
Logistic Regression	99.0% [96.5, 99.7]	0.991 [0.972, 1.000]	99.0% [94.6, 99.8]	99.0% [94.5, 99.8]	99.0%	99.0%
LDA	99.0% [96.5, 99.7]	0.993 [0.977, 1.000]	99.0% [94.6, 99.8]	99.0% [94.5, 99.8]	99.0%	99.0%
Random Forest	98.0% [95.0, 99.2]	0.994 [0.980, 1.000]	98.0% [93.1, 99.5]	98.0% [93.0, 99.5]	98.0%	98.0%
SVM (RBF)	99.0% [96.5, 99.7]	0.993 [0.973, 1.000]	99.0% [94.6, 99.8]	99.0% [94.5, 99.8]	99.0%	99.0%

PPV, positive predictive value; NPV, negative predictive value; AUC, area under the receiver operating characteristic curve. Male was treated as the positive class. Values in brackets are 95% confidence intervals (Wilson score method for accuracy, sensitivity and specificity; bootstrap percentile method for AUC).

**Table 11:** Classification performance on an independent 30% hold-out test set (n = 61).

Model	Test n	Accuracy	AUC	Sensitivity	Specificity
Logistic Regression	61	100.0% [94.1, 100.0]	1.000	100.0%	100.0%
LDA	61	100.0% [94.1, 100.0]	1.000	100.0%	100.0%
Random Forest	61	96.7% [88.8, 99.1]	0.999	96.8%	96.7%
SVM (RBF)	61	100.0% [94.1, 100.0]	1.000	100.0%	100.0%

Models were trained on a stratified 70% training set (n = 140) and evaluated on the independent 30% hold-out set (n = 61).

**Table 12:** Balanced accuracy, F1-score and Matthews correlation coefficient (MCC) for the four classifiers, under 10-fold cross-validation and on the independent hold-out set.

Model	Bal. Acc. (CV)	F1 (CV)	MCC (CV)	Bal. Acc. (Hold-Out)	F1 (Hold-Out)	MCC (Hold-Out)
Logistic Regression	99.0%	0.990	0.980	100.0%	1.000	1.000
LDA	99.0%	0.990	0.980	100.0%	1.000	1.000
Random Forest	98.0%	0.980	0.960	96.7%	0.968	0.934
SVM (RBF)	99.0%	0.990	0.980	100.0%	1.000	1.000

Bal. Acc., balanced accuracy; MCC, Matthews correlation coefficient.

**Table 13:** Stability of 10-fold cross-validated accuracy across 20 independently repeated random partitions.

Model	Mean CV Accuracy	SD	Range
Logistic Regression	98.83%	0.24	98.5–99.0%
LDA	99.00%	0.00	99.0–99.0%
Random Forest	98.03%	0.20	97.5–98.5%
SVM (RBF)	99.00%	0.00	99.0–99.0%

SD, standard deviation across the 20 repeats. Default classifier hyperparameters throughout (Section 2.5).

**Table 14:** Hyperparameter-optimised nested cross-validation accuracy for random forest and SVM, compared with the default-hyperparameter results of Table 9.

Model	Default (Table 9)	Nested-CV Optimised	Best Configuration
Random Forest	98.0%	98.02% (SD 2.55)	n_estimators = 100, max_depth = None, min_samples_leaf = 1
SVM (RBF)	99.0%	99.02% (SD 2.06)	C = 0.1, gamma = scale

Nested-CV optimised: mean accuracy (SD) across the outer 10-fold loop, with hyperparameters re-selected by grid search within each outer training fold. Best configuration: single best-scoring combination from a full grid search over the entire dataset (5-fold CV), reported for transparency; not used to generate any other result in this manuscript.

**Table 15:** Univariate discriminative power and sectioning points for the 14 pelvic measurements.

Variable	Univariate AUC	Sectioning Point (mm or °)	Accuracy at Cutoff
AW	0.972	50.9	92.5%
IL	0.961	104.0	91.0%
AH	0.958	51.2	90.5%
SPA	0.955	74.9	90.5%
AGN	0.950	80.8	88.6%
TPO	0.940	107.0	87.1%
IH	0.932	208.0	86.6%
PSL	0.805	34.4	74.1%
TPI	0.755	128.0	67.7%
IB	0.683	159.0	65.2%
PL	0.654	98.0	64.2%
TDSS	0.643	51.0	63.7%
AHS	0.606	122.0	60.7%
ABS	0.517	114.0	52.2%

AUC, area under the receiver operating characteristic curve. Sectioning points were derived using the Youden index. Accuracy at cutoff denotes the proportion of individuals correctly classified using the sectioning point alone.

**Table 10:** Pairwise McNemar’s exact test comparing cross-validated predictions of the four classifiers (n = 201).

Model 1	Model 2	Discordant Pairs (b, c)	McNemar Exact *p*
Logistic Regression	LDA	b = 0, c = 0	1.000
Logistic Regression	Random Forest	b = 2, c = 0	0.500
Logistic Regression	SVM (RBF)	b = 0, c = 0	1.000
LDA	Random Forest	b = 2, c = 0	0.500
LDA	SVM (RBF)	b = 0, c = 0	1.000
Random Forest	SVM (RBF)	b = 0, c = 2	0.500

Logistic regression, LDA, and SVM (RBF) produced identical predictions for every case under 10-fold cross-validation (0 discordant pairs for all three pairwise comparisons); random forest differed from each of the other three models on the same 2 cases. b, number of cases the first-listed model classified correctly and the second misclassified; c, the reverse.

**Table 16:** Bootstrap out-of-bag (OOB) validation of the Youden-derived sectioning point for each of the 14 pelvic measurements (1000 resamples).

Variable	Apparent Accuracy	Out-of-Bag Accuracy (Mean)	OOB 95% CI	Optimism
AW	92.5%	90.9%	[85.7, 95.8]	+1.6 pts
IL	91.0%	90.1%	[83.6, 95.8]	+1.0 pts
AH	90.5%	88.3%	[80.8, 94.2]	+2.3 pts
SPA	90.5%	89.1%	[82.5, 94.7]	+1.5 pts
AGN	88.6%	86.7%	[79.7, 92.8]	+1.8 pts
TPO	87.1%	85.7%	[78.6, 92.1]	+1.4 pts
IH	86.6%	84.8%	[77.9, 91.0]	+1.7 pts
PSL	74.1%	71.3%	[62.3, 79.0]	+2.9 pts
TPI	67.7%	66.4%	[57.7, 75.0]	+1.3 pts
IB	65.2%	62.3%	[53.6, 71.1]	+2.8 pts
PL	64.2%	62.7%	[53.2, 72.0]	+1.4 pts
TDSS	63.7%	60.8%	[52.1, 69.6]	+2.9 pts
AHS	60.7%	57.7%	[50.7, 66.7]	+3.0 pts
ABS	52.2%	54.2%	[50.0, 61.1]	−1.9 pts

Apparent accuracy is the same-sample classification accuracy at the Youden cutpoint (as in Table 15); OOB accuracy is the mean accuracy when the cutpoint re-derived on each bootstrap resample is applied to the corresponding out-of-bag cases; optimism is apparent minus mean OOB accuracy. A negative optimism value (ABS) indicates mean OOB accuracy slightly exceeded apparent accuracy, consistent with ABS performing near chance level (Table 15) and its Youden cutpoint being unstable across resamples rather than systematically overfitted.

## Data Availability

The data supporting the findings of this study are available from the corresponding author upon reasonable request. The data are not publicly available due to ethical and privacy restrictions.

## Supplementary Materials

The following supporting information can be downloaded at: https://www.mdpi.com/article/10.3390/diagnostics16182893/s1, Figure S1: Sex differences across all 14 pelvic measurements. Values are in millimetres, except SPA and AGN, which are in degrees; axis units are labelled per variable.
