# Supplementary material for: Machine Learning-Based Sex Estimation from Computed Tomography-Derived Pelvic Morphometric Measurements in a Contemporary Turkish Population
Source: Diagnostics (Basel). 2026 Sep 8;16(18):2893. doi: 10.3390/diagnostics16182893 (PMC13605450; doi:10.3390/diagnostics16182893)
Supplement: Supplementary file 1 [file diagnostics-16-02893-s001.zip › diagnostics-4515865-supplementary.pdf]

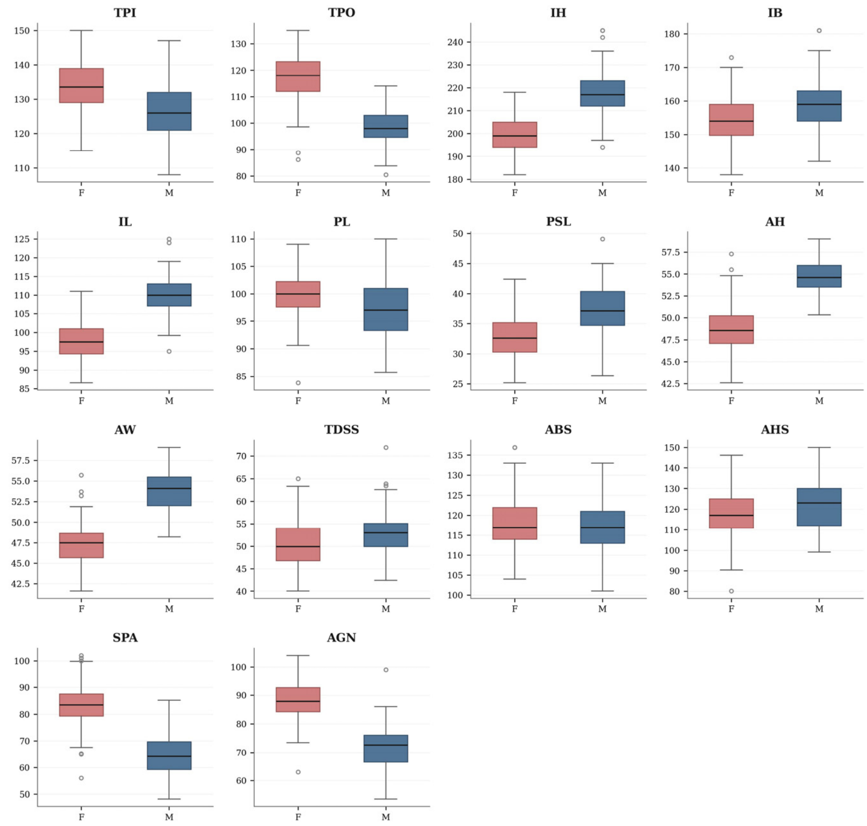

Figure S1. Sex differences across all 14 pelvic measurements. Values are in millimetres, except SPA and AGN, which are in degrees; axis units are labelled per variable.
